# The global prevalence of autism spectrum disorder: a comprehensive systematic review and meta-analysis

**DOI:** 10.1186/s13052-022-01310-w

**Published:** 2022-07-08

**Authors:** Nader Salari, Shabnam Rasoulpoor, Shna Rasoulpoor, Shamarina Shohaimi, Sima Jafarpour, Nasrin Abdoli, Behnam Khaledi-Paveh, Masoud Mohammadi

**Affiliations:** 1 Department of Biostatistics, School of Health, Kermanshah University of Medical Sciences, Kermanshah, Iran; 2 Department of Psychiatric Nursing, School of Nursing and Midwifery, Urmia University of Medical Sciences, Urmia, Iran; 3 Student research committee, Kermanshah University of Medical Sciences, Kermanshah, Iran; 4 Department of Biology, Faculty of Science, University Putra Malaysia, Serdang, Selangor Malaysia; 5 Department of Genetics and Molecular Biology, School of Medicine, Isfahan University of Medical Sciences, Isfahan, Iran; 6 Department of Psychiatry, Substance Abuse Prevention Research Center, Kermanshah University of Medical Sciences, Kermanshah, Iran; 7 Sleep Disorders Research Center, Kermanshah University of Medical Sciences, Kermanshah, Iran; 8 Cellular and Molecular Research Center, Gerash University of Medical Sciences, Gerash, Iran

**Keywords:** ASD, Autism spectrum disorder, Prevalence, systematic review, meta-analysis

## Abstract

**Background:**

Autism spectrum disorder (ASD) is one of the serious developmental disorders that is usually diagnosed below the age of three years. Although the severity of the disease’s symptoms varies from patient to patient, the ability to communicate with others is affected in all forms of ASD. This study aimed to determine the prevalence of ASD in high-risk groups by continent.

**Methods:**

The present study was conducted by systematic review and meta-analysis from 2008 to July 2021. Databases such as Science Direct, PubMed, Scopus, SID, Magiran, Web of Science (WoS), and Google Scholar from 2008 to July 2021 were searched to find related studies. Data were analysed using Comprehensive Meta-Analysis software (Version 2).

**Results:**

A total of 74 studies with 30,212,757 participants were included in this study. The prevalence of ASD in the world was 0.6% (95% confidence interval: 0.4–1%). Subgroup analyses indicated that the prevalence of ASD in Asia, America, Europe, Africa and Australia was 0.4% (95% CI: 0.1–1), 1% (95% CI: 0.8–1.1), 0.5% (95% CI: 0.2–1), 1% (95% CI: 0.3–3.1), 1.7% (95% CI: 0.5–6.1) respectively.

**Conclusion:**

ASD imposes a heavy health burden on communities around the world. Early detection of ASD can reduce the incidence of developmental disorders and improve patients’ communication skills. Therefore, health policymakers need to be aware of the prevalence and increasing trend of ASD to implement appropriate planning and interventions to reduce its consequences.

## Background

Autism Spectrum Disorder (ASD) is a neurological developmental disorder characterized by abnormalities in social relationships and repetitive or restricted behavioural patterns [1]. Numerous studies have been conducted on ASD, attributing the etiology of ASD to genetic, environmental, immunological, perinatal, neuroanatomical, and biochemical factors [2]. The autism spectrum encompasses a range of disorders, including Autistic disorder, Rett disorder, Asperger syndrome, and pervasive developmental disorder [3, 4].

Patients with ASD have deficits in social interactions, verbal and nonverbal social communication skills, as well as intelligence and motor functions. These patients also exhibit unusual interests, repetitive behaviours, and unusual responses to sensory experiences [5]. Autism spectrum disorder is associated with high levels of anxiety, stress, and isolation in patients’ families [6, 7]. Also, ASD imposes a heavy economic burden on society and the patients’ families [8]. These patients require considerable care, demanding significant financial resources. The direct and indirect costs of caring for children and adults with ASD in the United States in 2015 were estimated at $268.3 billion, which is more than the cost of stroke and hypertension. Overall, the cost of education, health care, and other lifelong services for an autistic patient varies from $ 1.4 million to $ 2.4 million per year [9].

Epidemiological surveys show an increasing trend in the annual prevalence of ASD. Besides the true increase in the prevalence of ASD, a variety of other reasons, such as a broader definition of ASD, changes in diagnostic criteria and screening tools, shifts in research methods, and increased awareness of ASD, have been suggested to contribute to this phenomenon [10–12].

Epidemiological studies have shown a rapid increase in the prevalence of ASD in recent years, with a prevalence of four to five times more in boys than girls. The average prevalence of autism spectrum disorder in Asia, Europe and North America is estimated at 1% [13, 14]. According to the Centers for Disease Control and Prevention (CDC) report in the United States, the prevalence of ASD among 8-year-old children was 1 in 59 in 2014 and 1 in 54 in 2016 [15]. The prevalence of ASD in children and adolescents in the United States was reported at 2.5% in 2014–2016 [16]. In another study in Italy, the prevalence of ASD among 7–9-year-old children was 1.15% [17]. In Asia, the prevalence of ASD has been reported to be 3.9%, with a prevalence of 0.14 to 2.9% in the Arab countries around the Persian Gulf [18, 19]. It is important to obtain an accurate estimation of the prevalence of autism to determine the economic burden and health services and allocate sufficient budget and services to autistic children or adults and their families [20]. In addition, by accurately determining the prevalence of ASD, vulnerable groups and geographical and environmental risk factors can be identified [21, 22].

This article provided an overall estimate of the global prevalence of ASD by systematically reviewing available studies. An updated and comprehensive estimate of the prevalence of autism spectrum disorder helps health professionals develop public health strategies. Therefore, given the importance of autism spectrum disorder, we conducted a systematic and meta-analysis of ASD prevalence studies worldwide.

## Methods

### Search strategy

This study was performed according to the Preferred Reporting Items for Systematic Reviews and Meta-Analyses (PRISMA 2020) guidelines [23]. Electronic databases such as Science Direct, PubMed, Scopus, SID, Magiran, Web of Science, and Google Scholar from 2008 to July 2021 were searched to find related studies. A comprehensive search was performed using the following keywords: autism, autistic disorder, ASD, autism spectrum conditions, epidemiology, cross-sectional study, and prevalence. All related studies were identified and transferred to EndNote software for reference selection management. Reference lists of related studies were also examined manually to find other potentially eligible studies.

### Inclusion and exclusion criteria

Studies were selected based on the following inclusion criteria: 1) Cross-sectional or cohort studies published from 2008 to 2021.2). 2) Articles were published in English and Persian. 4) Studies that used valid autism diagnostic tools such as: The Diagnostic and Statistical Manual of Mental Disorder,4th edition [DSM-IV], International Classification of Disease, 9th revision [ICD-9], Diagnostic and Statistical Manual of Mental Disorder text revision, 4th edition [DSM-IV TR], International Classification of Diseases, 10th revision [ICD-10], Diagnostic and Statistical Manual-5[DSM-5], or by tools (the Autism Diagnostic Interview Revised [ADI-R], Autism Diagnostic Observation Schedule [ADOS], Autism Behavior Checklist [ABC], Clancy Autism Behavior Scale [CABS], Children Autism Spectrum Test [CAST], and Checklist for Autism in Toddlers [CHAT]), Autism Spectrum Screening Questionnaire [ASSQ], Social Communication Disorder Checklist [SCDC], Modified Checklist for Autism in Toddlers [M-CHAT], Social Communication Questionnaire [SCQ], Indian Scale for Assessment of Autism [ISAA], Autism Quotient-10[AQ-10], Reporting Questionnaire for Children [RQC] 4) Studies that provided detailed information about participants and cases of autism spectrum disorder and its prevalence. The exclusion criteria were as follows: 1) Studies with duplicate or overlapping data; 2) studies without full text; 3) Studies with unknown detection methods.

### Study selection and data extraction

Initially, all databases were searched based on search strategies and duplicate studies were excluded. Subsequently, a list of relevant articles was prepared for further evaluation. In the first stage, the title and abstract of the remaining articles were carefully screened based on the inclusion and exclusion criteria. In the second stage, by evaluating the suitability of the studies, the full text of relevant articles remaining was examined, and irrelevant studies were excluded. To avoid bias, all steps were reviewed by two reviewers independently, and reasons for deleting articles were mentioned. In cases where there was disagreement between the two researchers, the article was reviewed by a third reviewer.

Information and characteristics of included articles such as the name of the first author, year of publication, type of residence, the origin of study, gender, sample size, age, assessment tool, diagnostic criteria, Autism Spectrum Disorder and the prevalence of ASD were extracted. Finally, a total of 74 articles were selected for quality assessment.

### Quality assessment

The methodological quality of studies was assessed according to the Reporting of Observational Studies in Epidemiology (STROBE) checklist. The STROBE checklist consisted of six scales / general sections, including title, abstract, introduction, methods, results, and discussion. Some of these scales had 32 items and included various methodological aspects of title, problem statement, study objectives, study type, the statistical population of the study, sampling method, determining the appropriate sample size, definition of variables and procedures, study data collection tools, statistical analysis methods and findings. The quality score ranged between 0 and 32; Studies with a score ≥ 16 were considered good and average methodological quality, and studies with a score <16 were identified as poor quality.

### Statistical analysis

I^2^ statistic test was used to evaluate the heterogeneity of selected studies. In order to assess the publication bias, due to the high volume of samples included in the studies, the Begg and Mazumdar test was used at a significance level of 0.1 and its corresponding Funnel plot. Data analysis was performed using Comprehensive Meta-Analysis software (Version 2).

## Result

### Search results and study characteristics

**Fig. 1:**
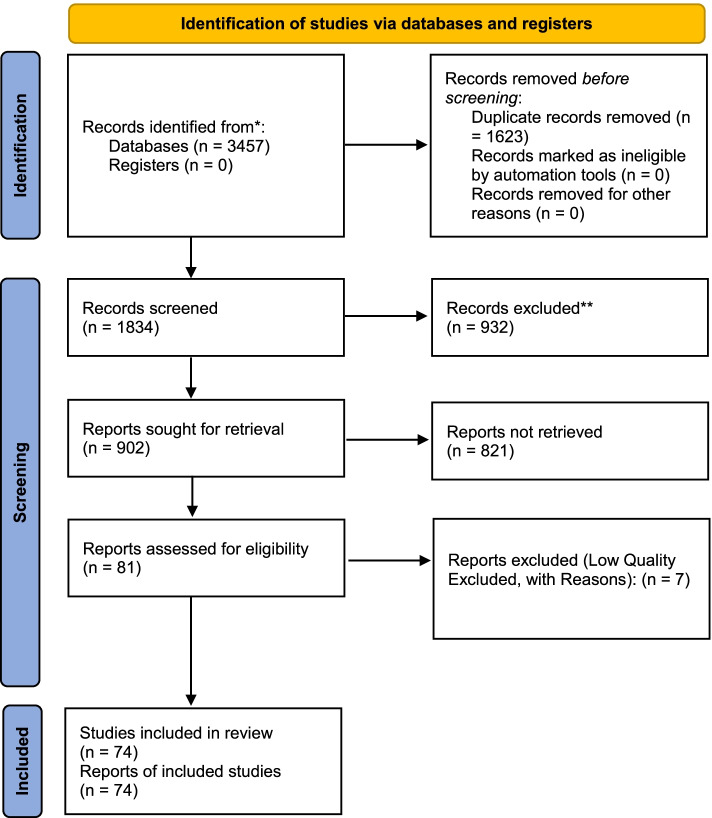
Flow chart indicating the stages of article selection in this review (PRISMA 2020)

A total of 3457 studies were collected in the initial literature search. After eliminating duplicate studies, 1834 eligible studies were selected by reviewing the titles and abstracts (Fig. 1). Of the 902 remaining studies in the screening stage, 821 articles were excluded by studying the full-text based on the inclusion and exclusion criteria. Seven articles were assessed as low quality and removed. Finally, a total of 74 articles were included in this meta-analysis. Studies were published from 2008 to 2021 in all countries of the world. A summary of the main findings and characteristics of the included studies are shown in Table 1. Differences in methods, the definition of autism, screening tools, and diagnostic criteria between countries made it very difficult to compare studies (Table 1 and Fig. 1).

**Table 1:** Summary of information and characteristics of selected articles for the systematic review

Year	Author	Country	Region	Sample size	Male (n)	Age (year)	Screening diagnostic tools	Diagnostic criteria	Prevalence (%)	Male with ASD(n)	Ref
Asia											
2018	Akhter	Bangladesh	Rural	5286	3436	1.5–3	M-CHAT ADOS	DS M-I V TR	4 (0.0 75)	3	[24]
2018	Heys	Nepal	Rural	4098	–	9–13	AQ-10	–	14 (0.342)	8	[25]
2017	Raina	India	Mixed	28,070	14,019	1–10	ISAA	–	43 (0.153)	23	[26]
2017	Rudra	India	Mixed	5947	3344	3–8	SCDC SCQ ADOS	–	13 (0.23)	11	[27]
2016	Chaaya	Lebanon	Urban	998	537	1.3–4	M-CHAT	–	15 (1.53)	8	[28]
2014	Jun Ping	China	Mixed	8000	4142	1.5–3	CHAT	DS M-I V	22 (0.275)	18	[29]
2015	Raz	Israel	–	2,431,649	–	8	–	DSM-IV-TR	9109 (0.37)	–	[30]
2016	Poovathinal	India	Mixed	18,480	9132	1–30	–	DSM-IV-TR	43 (0.23)	29	[31]
2015	Raina	India	Mixed	11,000	5243	1–10	ISAA	–	10 (0.09)	–	[32]
2016	Pantelis	Korean	–	23,234	–	7–12	ASSQ	–	553 (2.64)		[33]
2013	Davidovitch	Israeli	Mixed	423,524	218,076	1–12	–	DS M-I V	2034 (0.480)	1706	[34]
2012	Samadi	Iran	Mixed	1,320,334	78,701	5	SCQ ADI-R		826 (0.063)	669	[35]
2011	Chien	Taiwan	Mixed	372,642	185,420	0–17	–	DS M-I V TR	659 (0.177)	495 (75.0)	[36]
2011	Kim	Korean	Mixed	22,660	11,679	7–12	ASSQ ADOS ADI-R	DS M-I V	598 (2.639)	437	[37]
2009	Perera	Srilanka	mi-Urban	374	–	1.5–2	M-CHAT	DS M-I V	4 (1.07)	–	[38]
2015	Sun	China	–	714	371	6–10	CAST ADOS ADI-R	–	6 (0.85)	–	[39]
2009	Rabbani	Bangladesh	Mixed	3564	1763	5–17	RQC	DS M-I V TR	30 (0.842)	19 (63.3)	[40]
2011	Al-Farsi	Oman	Mixed	798,913	412,675	0–14	CARS	DSM-IV-TR	113 (0.014)	84	[41]
2011	Li	China	Mixed	616,940	–	0–17	–	ICD-10	77,301 (0.0238)	54,937	[42]
2019	Al-Mamri	Oman	–	837,655	426,450	0–14	–	DSM-5	1705 (0.2035)	1332	[43]
2019	Minh Hoang	Vietnam	Mixed	17,277	8009	1.5–2.5	M-CHAT	DSM-IV	130 (0.752)	107	[44]
2020	Zhou	China	Mixed	125,806	66,687	6–12	ADOS ADI-R	DSM-5	363 (0.29)	292	[45]
2019	Sun	China	Mixed	7167	3282	6–10	CAST ADOS ADI-R	DSM-IV-TR DSM-5	77 (1.08)	–	[46]
2018	Jin	China	Mixed	72,697	38,703	3–12	SCQ	DSM-5	203 (0.083)	157	[47]
2019	Alshaban	Qatar	Mixed	176,960	–	6–12	SCQ ADOS ADI-R	DSM-5	844 (1.14)	684	[44]
2013	Al-Zahrani	Saudi Arabia	–	22,950	–	7–12	ASSQ ADOS ADI-R	DSM-IV	8 (0.035)		[48]
American											
2009	Nicholas	USA	–	8156	–	4	–	DS M-I V TR	65 (0.8)	–	[49]
2009	Kogan	USA	–	77,911	40,405	3–17	–		913 (1.1)	746	[50]
2019	Imm	USA	–	152,259	–	8	–	DSM-IV-TR	1886 (1.24)	–	[21]
2018	Jon Baio	USA	Mixed	325,483	–	8	IQ	DSM-IV-TR	5473 (1.68)	–	[15]
2016	Christensen	USA	Mixed	346,978	–	8	IQ	DSM-IV-TR	5063 (1.46)		[51]
2017	Durkin	USA	–	1,308,641	668,575	8	–	DSM-IV-TR	13,396 (1.02)	11,033	[10]
2016	Fombonne	Mexico	–	4195	2074	8	LASI	DSM-IV-TR	36 (0.87)	–	[52]
2008	Nicholas	USA	–	47,726	–	8	–	DSM-IV-TR	295 (0.62)	–	[53]
2018	Diallo	Canada	–	1,447,660	–	1–17	–	ICD-10 ICD-9	16,940 (1.2)	–	[54]
2015	Dekkers	Ecuador	–	51,453	–	5–15	–	DSM-III	108 (0.21)	–	[55]
2008	Montiel-Nava	Venezuela	–	254,905	–	3–9	ADOS	DSM-IV-TR	430 (0.17)	–	[56]
2012	ADDM	USA	–	337,093	–	8	–	DSM-IV-TR ICD-9	3820 (1.13)	–	[57]
2019	Christensen	USA	–	70,887	–	4	–	DSM-IV-TR ICD-9	1208 (1.7)	–	[58]
2014	ADDM	USA	–	363,749	–	8	–	DSM-IV-TR ICD-9	5338 (1.47)	–	[59]
2020	Shaw	USA	–	72,277	–	4	–	DSM-IV-TR ICD-10 DSM-5	1125 (1.56)	–	[60]
2020	Maenner	USA	–	275,419	–	8	–	DSM-IV-TR DSM-5	5108 (1.85)	–	[61]
Africa											
2014	Lagunju	Nigeria	–	2320	–	1–10	–	DSM-IV	54 (2.3)	45	[62]
2014	Kakooza-Mwesige	Uganda	Mixed	1169	536	2–9	–	DSM-IV-TR	8 (0.68)	–	[63]
2012	Zeglam	Libya	–	38,508	–	0–16	–	DSM-IV	128 (0.33)		[64]
2016	Hewitt	Somali	–	12,329	6163	7–9	–	DSM-IV-TR	255 (2.07)	–	[65]
Europe											
2012	Kocovska	Faroe Islands		7128	3590	15–24	ASSQ ADOS	ICD-10 DS M-I V	67 (0.94)	49	[66]
2012	Nygren	Sweden	–	5007	–	2	M-CHAT	DSM-IV-TR	40 (0.80)	32	[67]
2018	Morales-Hidalgo	spain	–	2765	–	4–11	ADOS ADI-R	DSM-5	35 (1.26)	–	[68]
2010	Fernell	Sweden	Mixed	24,084	12,342	6	ADOS	ICD-10 DSM-IV-TR	147 (0.62)	123	[69]
2017	Skonieczna-Zydecka	Poland	Mixed	707,975	344,506	0–16	STAT ADOS Q-CHAT	ICD-10	2514 (0.35)	2038	[70]
2015	Idring	Sweden	–	735,096	376,617	0–27	–	ICD-10 DS M-I V	11,330 (1.54)	8033	[71]
2013	Saemundsen	Iceland	–	22,229	11,424	–	ADOS ADI-R	ICD-10 DS M-I V	267 (1.2)	197	[72]
2010	Posserud	Norway	–	6609	–	7–9	ASSQ ADOS ADI-R	ICD-10 DS M-I V	14 (0.21)	–	[73]
2012	Isaksen	Norway	Mixed	31,015	–	12	ADOS ADI-R	ICD-10	158 (0.51)	128	[74]
2011	Mattila	Finland	–	4422	–	8	ASSQ ADOS ADI-R FSIQ	DSM-IV-TR	37 (0.84)	–	[75]
2015	van Bakel	French		307,751		7	–	ICD-10	1123 (0.365)		[76]
2020	Narzisi	Italy	Mixed	10,138	5231	7–9	SCQ	DSM-5	81 (0.79)	68 (83.9)	[17]
2018	Bachmann	Germany	–	6,900,000 6,400,000	–	0–24	–	ICD-10	14,749 (0.22) 21,186 (0.38)	–	[77]
2009	Baron-Cohen	UK	Mixed	11,700		5–9	CAST ADOS ADI-R	ICD-10	83 (0.94)		[78]
2015	Stefan N. Hansen	Denmark	Mixed	677,915	347,955	0–20		ICD-8 ICD-10	3956 (0.579)	2865	[79]
2008	Parner	Denmark	Mixed	407,458	–	0–11	–	ICD-10	2649 (0.65)	–	[80]
2020	Thomaidis	Greece	–	182,879	93,897	10–11	–	ICD-10 DSM-5	2108 (1.15)	1715	[81]
2009	van Balkom	Island	–	13,109	–	0–9	ADOS	DSM-IV	69 (5.3)	–	[82]
2012	Pal Suren	Norway	–	731,318	–	0–11	–	DSM-IV	1415 (0.7)	–	[83]
2021	Fuentes	Spain	–	14,734	–	7–9	ADOS ADI-R SCQ	DSM-5	87 (0.59)	–	[84]
2016	Boilson	Ireland	–	5589	–	6–11	SCQ	–	44 (0.78)		[85]
2021	Linnsand	Sweden	–	902	454	2–5	ADOS	DSM-5	33 (3.66)	24	[86]
2013	Taylor	UK	–	256,278	132,143	2–8	–	DSM-IV	616 (0.24)	515	[87]
2008	Williams	UK	–	14,062	7111	11	–	ICD-10	86 (0.511)	75	[88]
Australia & New Zealand											
2020	May	Australia	Mixed	3381	1690	12–13	SDQ	–	145 (4.36)	111	[89]
2020	Bowden	New Zealand	Mixed	1,551,342	–	0–24	–	DSM-IV ICD-10	8955 (0.57)	–	[90]
2016	Randall	Australia	–	8366	4216	6–7	SDQ	–	165 (2)	136	[91]

*Abbreviation*: *CHAT* Checklist for Autism in Toddlers, *ASSQ* Autism Spectrum Screening Questionnaire, *ADOS* Autism Diagnostic Observation Schedule, *ADI-R* Autism Diagnostic Interview Revised, *SCDC* Social Communication Disorder Checklist, *M-CHAT* Modified Checklist for Autism in Toddlers, *SCQ* Social Communication Questionnaire, *ISAA* Indian Scale for Assessment of Autism, *AQ-10* Autism Quotient-10, *RQC* Reporting Questionnaire for Children, *DSM-IV* Diagnostic and Statistical Manual of Mental Disorder,4th edition, *ICD-9* International Classification of Disease, 9th revision, *ADOS* Autism Diagnostic Observation Schedule, *DSM-IV TR* Diagnostic and Statistical Manual of Mental Disorder text revision,4th edition, *ICD-10* International Classification of Diseases, 10th revision, *DSM-5* Diagnostic and Statistical Manual-5, *SDQ* Strengths and Difficulties Questionnaire

### Heterogeneity and publication bias

**Fig. 2:**
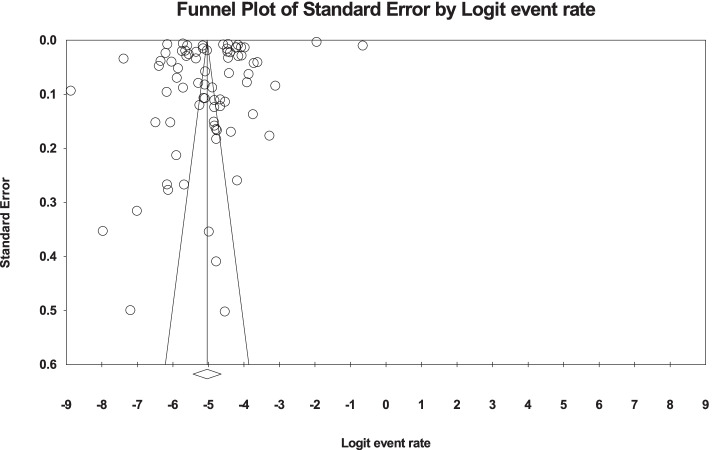
Funnel Plot Results related to the prevalence of ASD in the world

Egger’s and Begg’s tests were used to evaluate publication bias in the included studies. Results suggested no publication bias in the present study (*P* = 0.109) (Fig. 2). Based on the I^2^ test results (99.9%) and due to the heterogeneity of selected studies, the random-effects model was used to combine the reported results and estimate the prevalence of ASD. The potential reason for the heterogeneity between studies can be due to differences in the year of study, sample size, the origin of the study, and sampling error. The results were evaluated based on meta-regression.

**Fig. 3:**
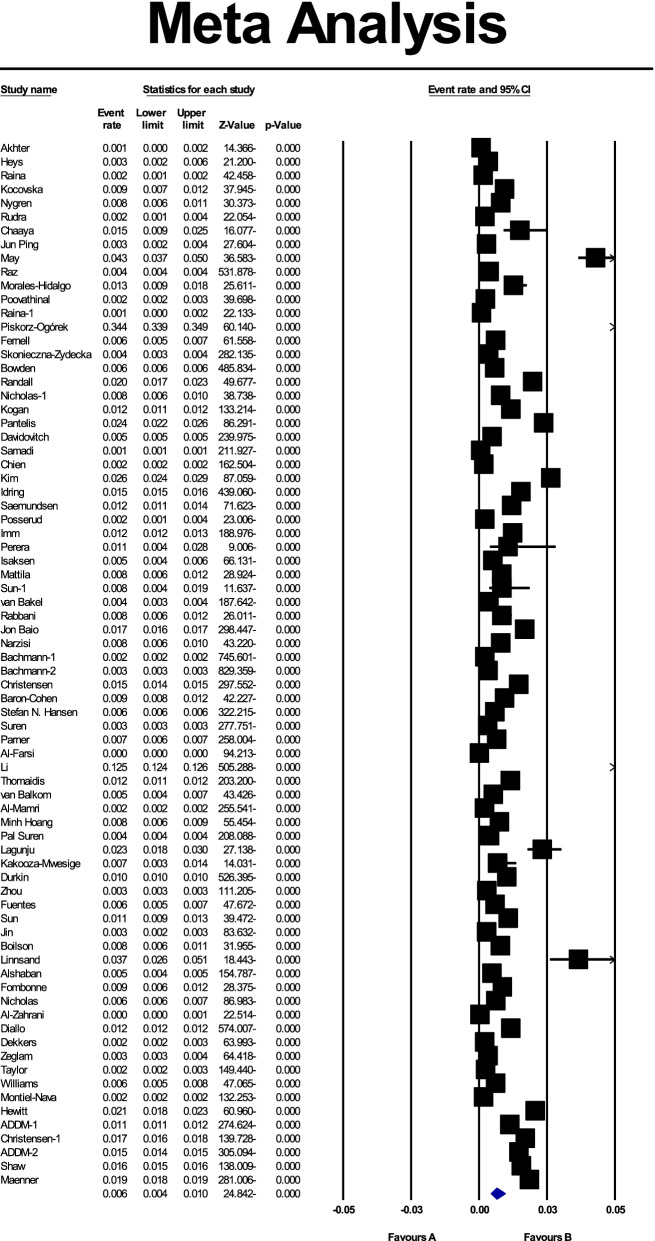
The prevalence of ASD in the world with 95% confidence interval

Based on the results of the present study, the global prevalence of ASD was 0.006 (95% CI: 0.004–0.01), or as a percentage of 0.6% (95% CI: 0.4–1),; the midpoint of each part shows the prevalence of each included study, and the diamond shape shows the prevalence of ASD in the population of all studies (Fig. 3).

### Meta-regression test

**Fig. 4:**
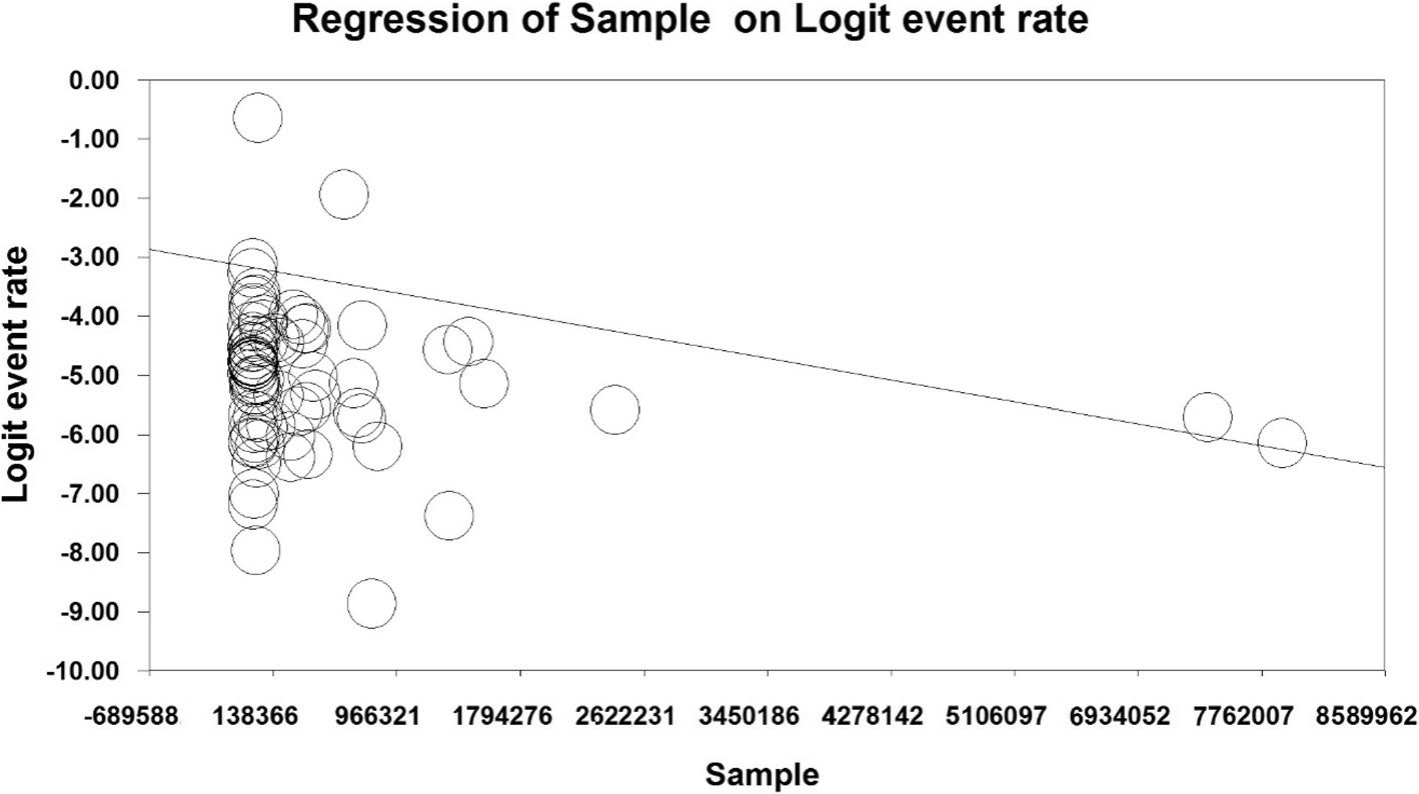
Meta-regression chart of the prevalence of ASD in the world by sample size

**Fig. 5:**
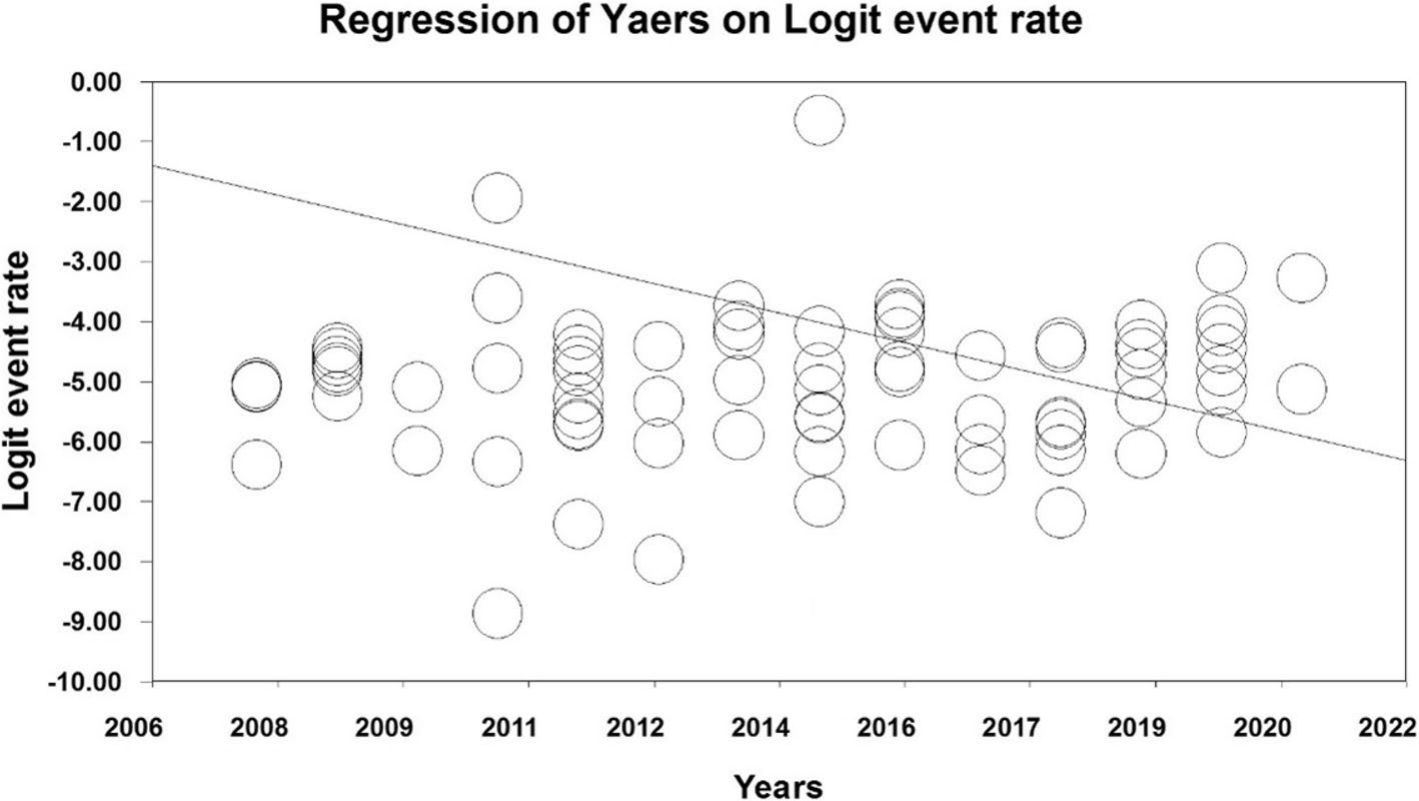
Meta-regression chart of the prevalence of ASD in the world by year

In order to investigate the effects of potential factors influencing the heterogeneity of the prevalence of ASD in the world, meta-regression was used for variables, including sample size and the year in which the study was conducted (Figs. 4 and 5). According to Fig. 4, the prevalence of ASD globally decreases with increasing sample size, which was statistically significant (*P* < 0.05) (Fig. 4). In addition, the prevalence of ASD in the world decreases with increasing the year in which the study was conducted (Fig. 5), and this was also statistically significant (*P* < 0.05).

### Subgroup analysis

Among the 74 studies, 26 were reviewed in Asia, 4 in Africa, 25 in Europe, 16 in the United States and 3 in Australia. The age of participants in these studies ranged from 0 to 27 years. the prevalence of ASD in Asia, America, Europe, Africa and Australia was 0.4% (95% CI: 0.1–1), 1% (95% CI: 0.8–1.1), 0.5% (95% CI: 0.2–1), 1% (95% CI: 0.3–3.1), 1.7% (95% CI: 0.5–6.1) respectively (Table 2) respectively (Table 2).

**Table 2:** Subgroup analysis of ASD prevalence by continents

Continent	Study (n)	Sample size	Heterogeneity I^2^%	Begg and Mazumdar Test	Prevalence (%) (95% CI)
Asia	26	7,356,939	99.9	0.103	0.4 (95% CI: 0.1–1)
America	16	3,758,240	99.6	0.137	1 (95% CI: 0.8–1.1)
Europe	25	17,480,163	99.9	0.171	0.5 (95% CI: 0.2–1)
Africa	4	54,326	99	0.734	1 (95% CI: 0.3–3.1)
Australia	3	1,563,089	99.7	0.296	1.7 (95% CI: 0.5–6.1)

## Discussion

In this study, we performed a systematic review and meta-analysis to provide a general and up-to-date estimate of the prevalence of ASD in different countries. A total of 74 cross-sectional and cohort studies were reviewed, and a total of 30,212,757 patients were assessed. We found that the prevalence of ASD varies from 0.02% in China to 3.66% in Sweden. The differences in estimating ASD prevalence were associated with research methods, screening tools, ASD definition and study population. The Prevalence of ASD in Asia, America, Europe, Africa and Australia was 0.4, 1, 0.5, 1, 1.7% respectively. According to our meta-analysis, the prevalence of ASD globally decreases by increasing the sample size in the world, which is consistent with previous studies [14] .

The prevalence of ASD worldwide has increased in recent decades [71, 78, 92]. Prevalence estimates also vary widely among studies from different countries, ranging from less than 0.2% in China and Italy to 2.7% in South Korea [37]. The differences in the prevalence of ASD are probably due to a number of reasons, including the fact that ASD is a spectral disease with different characteristics that even affect the definitions of ASD [93].

Other reasons for differences in the prevalence of ASD include different levels of awareness in various countries, cultural differences in interpreting children’s behaviours, variabilities in screening tools and diagnostic criteria, the lack of culture-sensitive diagnostic tools, the year of evaluation, and differences in sampling and studied populations (i.e., general population-based or hospital-based sampling) [37, 92]. Differences in study designs and protocols can affect the prevalence of ASD estimations, limiting the comparability of recent estimates [94].

Hansen et al. attributed 60% of the rising trend of ASD during recent years to alterations in diagnostic criteria and the incorporation of outpatients in the ASD registry [79]. Russell *et al.* emphasized the importance of improving diagnostic methods, increasing social awareness, and improving ASD-related behaviours by parents and teachers in the timely diagnosis and management of this condition [95]. A study in Sweden showed that ASD traits do not increase over time, but the number of children diagnosed with ASD increases, so it is concluded that changes in diagnostic tools may be responsible for the increased prevalence [96].

Our results suggested that differences in ASD prevalence can also be related to the geographical location of studied populations. In this regard, a higher prevalence in American and European countries compared to Asian countries is notable. In a cross-sectional study in Oman in 2011, the prevalence of ASD in children aged 0–14 years was estimated to be 0.14/1000. The low prevalence of ASD in Oman is probably due to underreporting and missed diagnoses [41].

In the latest study on Omani children, conducted from December 2011 to December 2018, the prevalence was reported as 2.04/1000, 15 times higher than the estimates in 2011. This increase can be attributed to improved diagnostic tools, increased awareness of ASD, better screening programs, and changes in diagnostic criteria. Even if it is almost 15 times higher than the previous study, it is still very low according to many estimates worldwide [43]. Moreover, the different prevalence of ASD in countries can be related to other socio-cultural and socio-economic factors [97–99].

According to our results, longitudinal analysis of the data of the same geographical region over the years confirmed an increase in the prevalence of ASD. For example, Randall et al. performed a longitudinal study on children in Australia in 2016 and estimated the prevalence of ASD as 14.1/1000 in 2005–2006 and 25.2/1000 in 2010–2011 [91], both of which were higher compared to a previously reported estimate (3.92/1000) [100]. In the most recent study in Australia in 2020, the prevalence of ASD was reported to be 43.6/1000 [89]. In another longitudinal study in Sweden on children aged 0–17 years, the prevalence of ASD was reported to increase from 4.2/1000 in 2001 to 14.4/1000 in 2011 [71].

In the United States, the Center for Disease Control and Prevention (CDC) established the ASD and Developmental Disabilities Monitoring Network (ADDM) in 2000 to screen children for ASD. The ADDM network provides the most up-to-date and comprehensive estimate of the prevalence of ASD and other growth disabilities in 8-year-old children, the age with the highest prevalence of ASD among children. Since 2010, the prevalence of ASD has also been estimated in 4-year-old children, and since 2000, these estimates have been updated bi-annually (the most recent estimates being related to 2016). The main advantage of this network is that it uses the same diagnostic criteria and follow-up methods for different groups of patients [15, 61, 101–103]. Although the prevalence of ASD in 8-year-old children in the United States increased from 6.7/1000 in 2000 to 11.3/1000 in 2008, it remained approximately unchanged from 2010 to 2012, but it started to increase again, reaching 16.8/1000 in 2014 and 18.5/1000 in the latest estimate in 2016 [15, 61, 103]. The same increasing trend in the prevalence of ASD has been observed in 4-year-old children, rising from 13.4/1000 in 2010 to 17/1000 in 2014 and descending to 15.6/1000 in 2016 [58, 60].

Qiu *et al*. conducted a systematic review and meta-analysis of 12 studies on the prevalence of ASD until August 2018 in Asia, reporting a widely variable prevalence among countries. Accordingly, ASD prevalence was estimated as 0.51, 0.31, and 0.35% in East, South, and West Asia. The studies showed that the prevalence of ASD was on the rise in Asia, with a higher prevalence in men than in women. According to these 12 studies, the overall prevalence of ASD in Asia was 0.36%, which was lower than the prevalence in Western countries [18] .

Our results show that there is very limited data on the prevalence of ASD in Africa compared to other parts of the world. Four studies on African communities (Uganda, Nigeria, Somalia, and Libya) were assessed in the present study [62–65]. These studies had estimated the prevalence of ASD in a mixed population from urban and rural regions, and most of them had used convenient sampling and extracted data from hospitals and specialized pediatric centres. A shortage in studies on ASD in African countries compared to other regions of the world may be explained by factors such as insufficient psychologists and psychiatrists and a lack of resources for and low interest in researching in this field [104, 105].

Gender is a prominent factor affecting ASD prevalence. According to the DSM-5, ASD in men is four times more common than in women [1]. Based on a comprehensive systematic review, the male to female ratio in children with ASD has been described as three to one [106], indicating a higher prevalence of ASD in males [92, 107, 108]. Nevertheless, some studies have reported similar ratios for males and females affected with ASD [109–112], which may be related to gender differences in presenting clinical symptoms. Generally, girls with ASD show fewer unusual behaviours and are less likely to be diagnosed with ASD [113]. Several studies based on clinical observations have shown that girls and women with ASD perform better in social communications and display fewer social and communication disorders than boys and men with ASD [114, 115].

Girls with ASD represent better speech behaviour and fewer abnormalities in communicational and social skills or show different repetitive and stereotyped activities than boys [116, 117]. These social and communication capabilities, which are related to a feature of the female phenotype, can help women adapt to social situations, masking some of the main symptoms of ASD and leading to either misdiagnosis or late diagnosis of ASD in girls [118, 119].

The age of diagnosis is another important factor in determining ASD prevalence. While ASD can be diagnosed at 24 months [120], various studies show that the age of diagnosis is from 36 to 120 months [121, 122]. The mean age is reported to be around 55 months [121]. and in milder forms such as Asperger syndrome, the diagnosis may be delayed until nine or even 11 years of age [88]. Nonetheless, severe ASD is usually diagnosed in the preschool years [123]. However, the severity and expression of ASD characteristics vary in patients with ASD, which can influence the time of diagnosis [124].

There are some limitations to our meta-analysis. First, different diagnostic tools and techniques in the included study may lead to selection bias. Second, the sample size was variable in the included studies, making it difficult to compare. Third, the number of studies was not available due to language limitations.

## Conclusion

The dramatic increase in ASD prevalence in recent years has been concerning. In developing countries, accurate and reliable estimates of ASD prevalence are needed so that public health experts and policymakers can develop strategic plans to meet patients’ needs. Early diagnosis and intervention can reduce ASD complications and related disabilities and improve educational performance and cognitive development in children suffering from ASD. Nonetheless, this study cannot draw a definite conclusion whether or not the increasing trend of ASD is real or is just due to altered diagnostic criteria and tools, leading to earlier and more diagnoses of ASD patients. Therefore, it is advisable to employ a common and consistent method in future studies. Because many studies could not be accessed due to language limitations, more research is needed to obtain more reliable information worldwide. There is no information on ASD prevalence in some countries, so more research is required to obtain such data for better global management of ASD.

## Data Availability

Datasets are available through the corresponding author upon reasonable request.

## Abbreviations

ASD: Autism spectrum disorder
WoS: Web of Science
DSM-IV: The Diagnostic and Statistical Manual of Mental Disorders,4th edition
ICD-9: International Classification of Disease, 9th revision
DSM-IV TR: Diagnostic and Statistical Manual of Mental Disorder text revision,4th edition
ICD-10: International Classification of Diseases, 10th revision
DSM-5: Diagnostic and Statistical Manual-5
ADI-R: Autism Diagnostic Interview Revised
ADOS: Autism Diagnostic Observation Schedule
ABC: Autism Behavior Checklist
CABS: Clancy Autism Behavior Scale
CAST: Children Autism Spectrum Test
CHAT: Checklist for Autism in Toddlers
ASSQ: Autism Spectrum Screening Questionnaire
SCDC: Social Communication Disorder Checklist
M-CHAT: Modified Checklist for Autism in Toddlers
SCQ: Social Communication Questionnaire
ISAA: Indian Scale for Assessment of Autism
AQ-10: Autism Quotient-10
RQC: Reporting Questionnaire for Children
STROBE: Strengthening the Reporting of Observational Studies in Epidemiology
PRISMA: Preferred Reporting Items for Systematic Reviews and Meta-Analysis

## References

[1] American Psychiatric Association A, Association AP (2013). Diagnostic and statistical manual of mental disorders: DSM-5.

[2] Pennington ML, Cullinan D, Southern LB. Defining autism: variability in state education agency definitions of and evaluations for autism spectrum disorders. Autism Res Treat. 2014;2014:327271. 10.1155/2014/327271 PMC4060325 24987527

[3] Taylor MJ, Rosenqvist MA, Larsson H, Gillberg C, D’Onofrio BM, Lichtenstein P (2020). Etiology of autism spectrum disorders and autistic traits over time. JAMA Psychiatry.

[4] Bölte S, Girdler S, Marschik PB (2019). The contribution of environmental exposure to the etiology of autism spectrum disorder. Cell Mol Life Sci.

[5] Lord C, Elsabbagh M, Baird G, Veenstra-Vanderweele J (2018). Autism spectrum disorder. Lancet.

[6] Bozkurt G, Uysal G, Düzkaya DS (2019). Examination of care burden and stress coping styles of parents of children with autism spectrum disorder. J Pediatr Nurs.

[7] Cohrs AC, Leslie DL (2017). Depression in parents of children diagnosed with autism spectrum disorder: a claims-based analysis. J Autism Dev Disord.

[8] Gordon-Lipkin E, Marvin AR, Law JK, Lipkin PH. Anxiety and mood disorder in children with autism spectrum disorder and ADHD. Pediatrics. 2018;141(4):e20171377. 10.1542/peds.2017-1377 29602900

[9] Leigh JP, Du J (2015). Brief report: Forecasting the economic burden of autism in 2015 and 2025 in the United States. J Autism Dev Disord.

[10] Durkin MS, Maenner MJ, Baio J, Christensen D, Daniels J, Fitzgerald R (2017). Autism spectrum disorder among US children (2002–2010): socioeconomic, racial, and ethnic disparities. Am J Public Health.

[11] Durkin MS, Wolfe BL (2020). Trends in autism prevalence in the US: A lagging economic indicator?. J Autism Dev Disord.

[12] Nevison CD, Blaxill M (2017). Diagnostic substitution for intellectual disability: a flawed explanation for the rise in autism. J Autism Dev Disord.

[13] Chiarotti F, Venerosi A (2020). Epidemiology of autism spectrum disorders: a review of worldwide prevalence estimates since 2014. Brain Sci.

[14] Fombonne E. Epidemiological controversies in autism. Swiss Archives of Neurology. Psychiatr Psychother. 2020;171: w03084

[15] Baio J, Wiggins L, Christensen DL, Maenner MJ, Daniels J, Warren Z (2018). Prevalence of autism spectrum disorder among children aged 8 years—autism and developmental disabilities monitoring network, 11 sites, United States, 2014. MMWR Surveill Summ.

[16] Xu G, Strathearn L, Liu B, Bao W (2018). Prevalence of autism spectrum disorder among US children and adolescents, 2014-2016. JAMA.

[17] Narzisi A, Posada M, Barbieri F, Chericoni N, Ciuffolini D, Pinzino M, et al. Prevalence of Autism Spectrum Disorder in a large Italian catchment area: a school-based population study within the ASDEU project. Epidemiol Psychiatr Sci. 2020;29:e5 10.1017/S2045796018000483 PMC8061252 30187843

[18] Qiu S, Lu Y, Li Y, Shi J, Cui H, Gu Y (2020). Prevalence of autism spectrum disorder in Asia: A systematic review and meta-analysis. Psychiatry Res.

[19] Alshaigi K, Albraheem R, Alsaleem K, Zakaria M, Jobeir A, Aldhalaan H (2020). Stigmatization among parents of autism spectrum disorder children in Riyadh, Saudi Arabia. Int J Pediatr Adolesc Med.

[20] Boswell K, Zablotsky B, Smith C (2014). Predictors of autism enrollment in public school systems. Except Child.

[21] Imm P, White T, Durkin MS (2019). Assessment of racial and ethnic bias in autism spectrum disorder prevalence estimates from a US surveillance system. Autism.

[22] Rice CE, Rosanoff M, Dawson G, Durkin MS, Croen LA, Singer A (2012). Evaluating changes in the prevalence of the Autism Spectrum Disorders (ASDs). Public Health Rev.

[23] Moher D, Shamseer L, Clarke M, Ghersi D, Liberati A, Petticrew M (2015). Preferred reporting items for systematic review and meta-analysis protocols (PRISMA-P) 2015 statement. Syst Rev.

[24] Akhter S, Hussain AE, Shefa J, Kundu GK, Rahman F, Biswas A. Prevalence of Autism Spectrum Disorder (ASD) among the children aged 18–36 months in a rural community of Bangladesh: a cross sectional study. F1000Research. 2018;7:424. 10.12688/f1000research.13563.1 PMC6039957 30026928

[25] Heys M, Gibbons F, Haworth E, Medeiros E, Tumbahangphe KM, Wickenden M (2018). The estimated prevalence of autism in school-aged children living in rural Nepal using a population-based screening tool. J Autism Dev Disord.

[26] Raina SK, Chander V, Bhardwaj AK, Kumar D, Sharma S, Kashyap V (2017). Prevalence of autism spectrum disorder among rural, urban, and tribal children (1–10 years of age). J Neurosci Rural Pract.

[27] Rudra A, Belmonte MK, Soni PK, Banerjee S, Mukerji S, Chakrabarti B (2017). Prevalence of autism spectrum disorder and autistic symptoms in a school-based cohort of children in Kolkata, India. Autism Res.

[28] Chaaya M, Saab D, Maalouf FT, Boustany R-M (2016). Prevalence of autism spectrum disorder in nurseries in Lebanon: a cross sectional study. J Autism Dev Disord.

[29] Huang JP, Cui SS, Yu H, Hertz-Picciotto I, Qi LH, Zhang X (2014). Prevalence and early signs of autism spectrum disorder (ASD) among 18–36 month old children in Tianjin of China. Biomed Environ Sci.

[30] Raz R, Weisskopf MG, Davidovitch M, Pinto O, Levine H (2015). Differences in autism spectrum disorders incidence by sub-populations in Israel 1992–2009: a total population study. J Autism Dev Disord.

[31] Poovathinal SA, Anitha A, Thomas R, Kaniamattam M, Melempatt N, Anilkumar A (2016). Prevalence of autism spectrum disorders in a semiurban community in south India. Ann Epidemiol.

[32] Raina SK, Kashyap V, Bhardwaj AK, Kumar D, Chander V (2015). Prevalence of autism spectrum disorders among children (1-10 years of age) - findings of a mid-term report from Northwest India. J Postgrad Med.

[33] Pantelis PC, Kennedy DP (2016). Estimation of the prevalence of autism spectrum disorder in South Korea, revisited. Autism.

[34] Davidovitch M, Hemo B, Manning-Courtney P, Fombonne E (2013). Prevalence and incidence of autism spectrum disorder in an Israeli population. J Autism Dev Disord.

[35] Samadi SA, Mahmoodizadeh A, McConkey R (2012). A national study of the prevalence of autism among five-year-old children in Iran. Autism.

[36] Chien I-C, Lin C-H, Chou Y-J, Chou P (2011). Prevalence and incidence of autism spectrum disorders among national health insurance enrollees in Taiwan from 1996 to 2005. J Child Neurol.

[37] Kim YS, Leventhal BL, Koh Y-J, Fombonne E, Laska E, Lim E-C (2011). Prevalence of autism spectrum disorders in a total population sample. Am J Psychiatr.

[38] Perera H, Wijewardena K, Aluthwelage R (2009). Screening of 18–24-month-old children for autism in a semi-urban community in Sri Lanka. J Trop Pediatr.

[39] Sun X, Allison C, Matthews FE, Zhang Z, Auyeung B, Baron-Cohen S (2015). Exploring the Underdiagnosis and Prevalence of Autism Spectrum Conditions in Beijing. Autism Res.

[40] Jahan N, Rahman A, Choudhury S, Chowdhury K, Wahab M, Rahman F (2009). Prevalence of mental disorders, mental retardation, epilepsy and substance abuse in children.

[41] Al-Farsi YM, Al-Sharbati MM, Al-Farsi OA, Al-Shafaee MS, Brooks DR, Waly MI (2011). Brief report: Prevalence of autistic spectrum disorders in the Sultanate of Oman. J Autism Dev Disord.

[42] Li N, Chen G, Song X, Du W, Zheng X (2011). Prevalence of autism-caused disability among Chinese children: a national population-based survey. Epilepsy Behav.

[43] Al-Mamri W, Idris AB, Dakak S, Al-Shekaili M, Al-Harthi Z, Alnaamani AM (2019). Revisiting the prevalence of autism spectrum disorder among Omani children: a multicentre study. Sultan Qaboos Univ Med J.

[44] Alshaban F, Aldosari M, Al-Shammari H, El-Hag S, Ghazal I, Tolefat M (2019). Prevalence and correlates of autism spectrum disorder in Qatar: a national study. J Child Psychol Psychiatry.

[45] Zhou H, Xu X, Yan W, Zou X, Wu L, Luo X (2020). Prevalence of autism spectrum disorder in China: a nationwide multi-centre population-based study among children aged 6 to 12 years. Neurosci Bull.

[46] Sun X, Allison C, Wei L, Matthews FE, Auyeung B, Wu YY (2019). Autism prevalence in China is comparable to Western prevalence. Mol Autism.

[47] Jin Z, Yang Y, Liu S, Huang H, Jin X (2018). Prevalence of DSM-5 autism spectrum disorder among school-based children aged 3–12 years in Shanghai, China. J Autism Dev Disord.

[48] Al-Zahrani A (2013). Prevalence and clinical characteristics of autism spectrum disorders in school-age children in Taif-KSA. Int J Med Sci Public Health.

[49] Nicholas JS, Carpenter LA, King LB, Jenner W, Charles JM (2009). Autism spectrum disorders in preschool-aged children: prevalence and comparison to a school-aged population. Ann Epidemiol.

[50] Kogan MD, Blumberg SJ, Schieve LA, Boyle CA, Perrin JM, Ghandour RM (2009). Prevalence of parent-reported diagnosis of autism spectrum disorder among children in the US, 2007. Pediatrics.

[51] Christensen D, Braun K, Baio J, Bilder D, Charles J, Constantino J, Lee LC (2016). Prevalence and characteristics of autism spectrum disorder among children aged 8 years—autism and developmental disabilities monitoring network, 11 sites, United States, 2012. MMWR Surveillance Summaries.

[52] Fombonne E, Marcin C, Manero AC, Bruno R, Diaz C, Villalobos M (2016). Prevalence of autism spectrum disorders in Guanajuato, Mexico: the leon survey. J Autism Dev Disord.

[53] Nicholas JS, Charles JM, Carpenter LA, King LB, Jenner W, Spratt EG (2008). Prevalence and characteristics of children with autism-spectrum disorders. Ann Epidemiol.

[54] Diallo FB, Fombonne É, Kisely S, Rochette L, Vasiliadis H-M, Vanasse A (2018). Prevalence and correlates of autism spectrum disorders in Quebec: Prévalence et corrélats des troubles du spectre de l’autisme au Québec. Can J Psychiatr.

[55] Dekkers LM, Groot NA, Mosquera END, Zúniga IPA, Delfos MF (2015). Prevalence of autism spectrum disorders in Ecuador: a pilot study in Quito. J Autism Dev Disord.

[56] Montiel-Nava C, Peña JA (2008). Epidemiological findings of pervasive developmental disorders in a Venezuelan study. Autism.

[57] Autism, Investigators DDMNSYP (2012). Prevalence of autism spectrum disorders—autism and developmental disabilities monitoring network, 14 sites, United States, 2008. MMWR Surveill Summ.

[58] Christensen DL, Maenner MJ, Bilder D, Constantino JN, Daniels J, Durkin MS (2019). Prevalence and characteristics of autism spectrum disorder among children aged 4 years—early autism and developmental disabilities monitoring network, seven sites, United States, 2010, 2012, and 2014. MMWR Surveill Summ.

[59] Autism, Investigators DDMNSYP (2014). Prevalence of autism spectrum disorder among children aged 8 years—autism and developmental disabilities monitoring network, 11 sites, United States, 2010. MMWR Surveill Summ.

[60] Shaw KA, Maenner MJ, Baio J (2020). Early identification of autism spectrum disorder among children aged 4 years—Early Autism and Developmental Disabilities Monitoring Network, six sites, United States, 2016. MMWR Surveill Summ.

[61] Maenner MJ, Shaw KA, Baio J (2020). Prevalence of autism spectrum disorder among children aged 8 years—autism and developmental disabilities monitoring network, 11 sites, United States, 2016. MMWR Surveill Summ.

[62] Lagunju I, Bella-Awusah T, Omigbodun O (2014). Autistic disorder in Nigeria: profile and challenges to management. Epilepsy Behav.

[63] Kakooza-Mwesige A, Ssebyala K, Karamagi C, Kiguli S, Smith K, Anderson MC (2014). Adaptation of the “ten questions” to screen for autism and other neurodevelopmental disorders in Uganda. Autism.

[64] Zeglam A, Maouna A. Prevalence of autistic spectrum disorders in Tripoli, Libya: the need for more research and planned services. East Mediterr Health J. 2012;18(2):184-8. 10.26719/2012.18.2.184 22571097

[65] Hewitt A, Hall-Lande J, Hamre K, Esler AN, Punyko J, Reichle J (2016). Autism spectrum disorder (ASD) prevalence in Somali and non-Somali children. J Autism Dev Disord.

[66] Kočovská E, Biskupstø R, Gillberg IC, Ellefsen A, Kampmann H, Stórá T (2012). The rising prevalence of autism: a prospective longitudinal study in the Faroe Islands. J Autism Dev Disord.

[67] Nygren G, Cederlund M, Sandberg E, Gillstedt F, Arvidsson T, Gillberg IC (2012). The prevalence of autism spectrum disorders in toddlers: a population study of 2-year-old Swedish children. J Autism Dev Disord.

[68] Morales-Hidalgo P, Roigé-Castellví J, Hernández-Martínez C, Voltas N, Canals J (2018). Prevalence and characteristics of autism spectrum disorder among Spanish school-age children. J Autism Dev Disord.

[69] Fernell E, Gillberg C (2010). Autism spectrum disorder diagnoses in Stockholm preschoolers. Res Dev Disabil.

[70] Skonieczna-Żydecka K, Gorzkowska I, Pierzak-Sominka J, Adler G (2017). The prevalence of autism spectrum disorders in West Pomeranian and Pomeranian regions of Poland. J Appl Res Intellect Disabil.

[71] Idring S, Lundberg M, Sturm H, Dalman C, Gumpert C, Rai D (2015). Changes in prevalence of autism spectrum disorders in 2001–2011: findings from the Stockholm youth cohort. J Autism Dev Disord.

[72] Saemundsen E, Magnússon P, Georgsdóttir I, Egilsson E, Rafnsson V. Prevalence of autism spectrum disorders in an Icelandic birth cohort. BMJ Open. 2013;3(6):e002748. 10.1136/bmjopen-2013-002748 PMC3693420 23788511

[73] Posserud M, Lundervold AJ, Lie SA, Gillberg C (2010). The prevalence of autism spectrum disorders: impact of diagnostic instrument and non-response bias. Soc Psychiatry Psychiatr Epidemiol.

[74] Isaksen J, Diseth TH, Schjølberg S, Skjeldal OH (2012). Observed prevalence of autism spectrum disorders in two Norwegian counties. Eur J Paediatr Neurol.

[75] Mattila M-L, Kielinen M, Linna S-L, Jussila K, Ebeling H, Bloigu R (2011). Autism spectrum disorders according to DSM-IV-TR and comparison with DSM-5 draft criteria: an epidemiological study. J Am Acad Child Adolesc Psychiatry.

[76] van Bakel MME, Delobel-Ayoub M, Cans C, Assouline B, Jouk P-S, Raynaud J-P (2015). Low but increasing prevalence of autism spectrum disorders in a French area from register-based data. J Autism Dev Disord.

[77] Bachmann CJ, Gerste B, Hoffmann F (2018). Diagnoses of autism spectrum disorders in Germany: time trends in administrative prevalence and diagnostic stability. Autism.

[78] Baron-Cohen S, Scott FJ, Allison C, Williams J, Bolton P, Matthews FE (2009). Prevalence of autism-spectrum conditions: UK school-based population study. Br J Psychiatry.

[79] Hansen SN, Schendel DE, Parner ET (2015). Explaining the increase in the prevalence of autism spectrum disorders: the proportion attributable to changes in reporting practices. JAMA Pediatr.

[80] Parner ET, Schendel DE, Thorsen P (2008). Autism prevalence trends over time in Denmark: changes in prevalence and age at diagnosis. Arch Pediatr Adolesc Med.

[81] Thomaidis L, Mavroeidi N, Richardson C, Choleva A, Damianos G, Bolias K (2020). Autism Spectrum Disorders in Greece: Nationwide Prevalence in 10–11 Year-Old Children and Regional Disparities. J Clin Med.

[82] Van Balkom ID, Bresnahan M, Vogtländer MF, van Hoeken D, Minderaa RB, Susser E (2009). Prevalence of treated autism spectrum disorders in Aruba. J Neurodev Disord.

[83] Surén P, Bakken IJ, Aase H, Chin R, Gunnes N, Lie KK (2012). Autism spectrum disorder, ADHD, epilepsy, and cerebral palsy in Norwegian children. Pediatrics.

[84] Fuentes J, Basurko A, Isasa I, Galende I, Muguerza MD, García-Primo P (2021). The ASDEU autism prevalence study in northern Spain. Eur Child Adolesc Psychiatry.

[85] Boilson A, Staines A, Ramirez A, Posada M, Sweeney M (2016). Operationalisation of the European Protocol for Autism Prevalence (EPAP) for autism spectrum disorder prevalence measurement in Ireland. J Autism Dev Disord.

[86] Linnsand P, Gillberg C, Nilses Å, Hagberg B, Nygren G (2021). A high prevalence of autism spectrum disorder in preschool children in an immigrant, multiethnic population in Sweden: challenges for health care. J Autism Dev Disord.

[87] Taylor B, Jick H, MacLaughlin D. Prevalence and incidence rates of autism in the UK: time trend from 2004–2010 in children aged 8 years. BMJ Open. 2013;3(10):e003219. 10.1136/bmjopen-2013-003219 PMC3808754 24131525

[88] Williams E, Thomas K, Sidebotham H, Emond A (2008). Prevalence and characteristics of autistic spectrum disorders in the ALSPAC cohort. Dev Med Child Neurol.

[89] May T, Brignell A, Williams K (2020). Autism spectrum disorder prevalence in children aged 12–13 years from the longitudinal study of Australian children. Autism Res.

[90] Bowden N, Thabrew H, Kokaua J, Audas R, Milne B, Smiler K (2020). Autism spectrum disorder/Takiwātanga: An Integrated Data Infrastructure-based approach to autism spectrum disorder research in New Zealand. Autism.

[91] Randall M, Sciberras E, Brignell A, Ihsen E, Efron D, Dissanayake C (2016). Autism spectrum disorder: Presentation and prevalence in a nationally representative Australian sample. Aus N Z J Psychiatry.

[92] Elsabbagh M, Divan G, Koh YJ, Kim YS, Kauchali S, Marcín C (2012). Global prevalence of autism and other pervasive developmental disorders. Autism Res.

[93] American Psychiatric Association A (1980). Diagnostic and statistical manual of mental disorders: American Psychiatric Association Washington, DC.

[94] Fombonne E (2009). Epidemiology of pervasive developmental disorders. Pediatr Res.

[95] Russell G, Collishaw S, Golding J, Kelly SE, Ford T (2015). Changes in diagnosis rates and behavioural traits of autism spectrum disorder over time. BJPsych Open.

[96] Lundström S, Reichenberg A, Anckarsäter H, Lichtenstein P, Gillberg C. Autism phenotype versus registered diagnosis in Swedish children: prevalence trends over 10 years in general population samples. BMJ. 2015;350:h1961. 10.1136/bmj.h1961 PMC4413835 25922345

[97] La Roche MJ, Bush HH, D'Angelo E (2018). The assessment and treatment of autism spectrum disorder: A cultural examination. Pract Innov.

[98] de Leeuw A, Happé F, Hoekstra RA (2020). A conceptual framework for understanding the cultural and contextual factors on autism across the globe. Autism Res.

[99] Taiwo T (2019). Organophosphate Exposures, Financial Hardship and Child Neurodevelopmental Outcomes in the CHARGE study. Environ Epidemiolo.

[100] Icasiano F, Hewson P, Machet P, Cooper C, Marshall A (2004). Childhood autism spectrum disorder in the Barwon region: a community-based study. J Paediatr Child Health.

[101] Rice C (2009). Prevalence of autism spectrum disorders--Autism and developmental disabilities monitoring network, United States, 2006.

[102] Control CD (2007). Prevention. Prevalence of autism spectrum disorders--autism and developmental disabilities monitoring network, 14 sites, United States, 2002. MMWR Surveill Summ (Washington, DC: 2002).

[103] Christensen DL, Braun KVN, Baio J, Bilder D, Charles J, Constantino JN (2018). Prevalence and characteristics of autism spectrum disorder among children aged 8 years—autism and developmental disabilities monitoring network, 11 sites, United States, 2012. MMWR Surveill Summ.

[104] Ruparelia K, Abubakar A, Badoe E, Bakare M, Visser K, Chugani DC (2016). Autism spectrum disorders in Africa: current challenges in identification, assessment, and treatment: a report on the International Child Neurology Association Meeting on ASD in Africa, Ghana, April 3-5, 2014. J Child Neurol.

[105] Abubakar A, Ssewanyana D, Newton CR. A systematic review of research on autism spectrum disorders in Sub-Saharan Africa. Behav Neurol. 2016;2016:3501910. 10.1155/2016/3501910 PMC5107214 27872512

[106] Loomes R, Hull L, Mandy WPL (2017). What is the male-to-female ratio in autism spectrum disorder? A systematic review and meta-analysis. J Am Acad Child Adolesc Psychiatry.

[107] Hiller RM, Young RL, Weber N (2014). Sex differences in autism spectrum disorder based on DSM-5 criteria: evidence from clinician and teacher reporting. J Abnorm Child Psychol.

[108] Investigators P, Control CfD, Prevention (2014). Prevalence of autism spectrum disorder among children aged 8 years-autism and developmental disabilities monitoring network, 11 sites, United States, 2010. MMWR Surveill Summ (Washington, DC: 2002).

[109] Harrop C, Gulsrud A, Kasari C (2015). Does gender moderate core deficits in ASD? An investigation into restricted and repetitive behaviors in girls and boys with ASD. J Autism Dev Disord.

[110] Mussey JL, Ginn NC, Klinger LG (2017). Are males and females with autism spectrum disorder more similar than we thought?. Autism.

[111] Messinger DS, Young GS, Webb SJ, Ozonoff S, Bryson SE, Carter A (2015). Early sex differences are not autism-specific: A Baby Siblings Research Consortium (BSRC) study. Mol Autism.

[112] Sutherland R, Hodge A, Bruck S, Costley D, Klieve H (2017). Parent-reported differences between school-aged girls and boys on the autism spectrum. Autism.

[113] Guerra S, Spoto A, Castiello U, Parma V (2019). Sex differences in body ownership in adults with autism spectrum disorder. Front Psychol.

[114] Rivet TT, Matson JL (2011). Review of gender differences in core symptomatology in autism spectrum disorders. Res Autism Spectr Disord.

[115] Werling DM, Geschwind DH (2013). Sex differences in autism spectrum disorders. Curr Opin Neurol.

[116] Head AM, McGillivray JA, Stokes MA (2014). Gender differences in emotionality and sociability in children with autism spectrum disorders. Mol Autism.

[117] Lai M-C, Lombardo MV, Auyeung B, Chakrabarti B, Baron-Cohen S (2015). Sex/gender differences and autism: setting the scene for future research. J Am Acad Child Adolesc Psychiatry.

[118] Hiller RM, Young RL, Weber N (2016). Sex differences in pre-diagnosis concerns for children later diagnosed with autism spectrum disorder. Autism.

[119] Dworzynski K, Ronald A, Bolton P, Happé F (2012). How different are girls and boys above and below the diagnostic threshold for autism spectrum disorders?. J Am Acad Child Adolesc Psychiatry.

[120] Zwaigenbaum L, Bryson S, Garon N (2013). Early identification of autism spectrum disorders. Behav Brain Res.

[121] Brett D, Warnell F, McConachie H, Parr JR (2016). Factors affecting age at ASD diagnosis in UK: no evidence that diagnosis age has decreased between 2004 and 2014. J Autism Dev Disord.

[122] Daniels AM, Mandell DS (2014). Explaining differences in age at autism spectrum disorder diagnosis: a critical review. Autism.

[123] Wiggins LD, Baio J, Rice C (2006). Examination of the time between first evaluation and first autism spectrum diagnosis in a population-based sample. J Dev Behav Pediatr.

[124] Barbaro J, Dissanayake C (2009). Autism spectrum disorders in infancy and toddlerhood: a review of the evidence on early signs, early identification tools, and early diagnosis. J Dev Behav Pediatr.

